# Nurses' Working Conditions: Implications for Infectious Disease^1^

**DOI:** 10.3201/eid1011.040253

**Published:** 2004-11

**Authors:** Patricia W. Stone, Sean P. Clarke, Jeannie Cimiotti, Rosaly Correa-de-Araujo

**Affiliations:** * Columbia University School of Nursing, New York, New York, USA;; † University of Pennsylvania School of Nursing, Philadelphia, Pennsylvania, USA;; ‡ Agency for Healthcare Research and Quality, Rockville, Maryland, USA

**Keywords:** nurse working conditions, patient safety, healthcare-associated infections, occupational injuries, emerging infectious disease, synopsis

## Abstract

Poor working conditions are associated with risk for occupational infections.

Staffing patterns and nurses' working conditions are risk factors for healthcare-associated infections as well as occupational injuries and infections. Staffing shortages, especially of nurses, have been identified as one of the major factors expected to constrain hospitals' ability to deal with future outbreaks of emerging infections. These problems are compounded by a global nursing shortage. Understanding and improving nurses' working conditions can potentially decrease the incidence of many infectious diseases. Relevant research is reviewed, and policy options are discussed.

The Institute of Medicine's report, To Err is Human, which spotlighted the problem of patient safety, reported that tens of thousands of Americans die each year as a result of human error in the delivery of health care (*1*). Authors of a more recent Institute of Medicine report, Keeping Patients Safe, Transforming the Work Environment of Nurses, concluded that nursing is inseparably linked to patient safety and emphasized that poor working conditions for nurses and inadequate nurse staffing levels increase the risk for errors (*2*). Nurse working conditions are related to patients' risk of healthcare-associated infections and occupational injuries and infections among staff (*3*). We discuss the nurse workforce, review research examining nursing as it relates to infectious disease, identify gaps in the literature, and discuss potential policy options. Although our focus is on the nursing workforce in the United States, international trends and comparisons are also discussed.

## The Nursing Workforce

Nearly 3 million registered nurses (RNs) work in the United States. Ninety-five percent of these nurses are women, as are most of the 700,000 licensed practical nurses and >2 million unlicensed nurse assistants. Internationally, occupational distributions are similar.

More than 1 million RNs work in hospitals, which makes nursing the largest hospital workforce. In 60% of U.S. hospitals, vacancy rates for RNs have increased since 1999; 14% of hospitals now report a severe nurse shortage (i.e., >20% of positions vacant). The American Hospital Association has reported that hospitals have up to 168,000 vacant positions; 126,000 (75%) of the available positions in these hospitals are for RNs (*4*). The current nursing shortage is related to an aging workforce, problems with retaining licensed personnel, and difficulty recruiting young people into the nursing workforce. The demand for RNs is projected to grow by 22% by 2008, and unless market corrections are made, the nursing shortage may reach 800,000 vacant positions by 2020 (*5*). Recent reports document that the nursing shortage is a severe and growing global problem (*4*).

Historically, the turnover rate among nurses is more than double that for other professionals of comparable education and sex (*6*). Recent estimates in U.S. hospitals of RN turnover and intention to quit have ranged from 17% to 36% (*6**,**7*), figures that compare to an overall turnover rate of 2.2% for those employed in health services and social services and 1.2% for those employed in educational services. In an investigation of the effects of various nurse working conditions in intensive care units, researchers found >17% of RNs indicated their intentions to quit within 1 year (P.W. Stone, unpub. data). This finding was disconcerting because this national U.S. sample of 2,324 RNs was highly qualified; their average experience in health care was 15.6 years (SD = 9.20), and their average tenure in their current position was 8.0 years (SD = 7.50). Of those intending to leave, 72% expressed poor working conditions as the reason. In an American Hospital Association–sponsored study, researchers estimated the cost of replacing one RN to be $30,000–$64,000 (*4*).

To cover patient census fluctuations and unplanned absences and to fill vacant positions caused by this nursing shortage, many healthcare facilities have increased nurses' patient loads or expanded the use of nonpermanent staff, such as float pool and agency nurses (*4*). Concerns have been voiced that reliance on agency nursing services elevates hospital costs, increases the fragmentation of health care, and discourages longer term proactive solutions to staffing shortages that would improve the morale of the permanent staff as well as the quality of patient care services (*8*). Extended work shifts and overtime for nurses have also escalated; however, nurses report making more errors when working shifts >12 hours, working overtime, or working >40 hours per week (*9*).

To increase the overall supply of nurses, many countries are increasingly relying on international recruitment and migration (*10*). The percentage of foreign-trained nurses in the United States is 4%, compared to 8% in the United Kingdom and 23% in New Zealand (*11*). However, the actual number of foreign-trained nurses in the United States is 90,000, which compares to 42,000 in the United Kingdom (*12*). In 2002, for the first time more foreign-trained nurses (n = 16,155) were newly registered in Britain than were those who had been educated within the country (n = 14,538). Many concerns exist about clinical competencies, cultural sensitivity, and ethics of the practice of importing nurses (*13*). While international recruitment can be a solution in one country, it can create additional shortages in others.

## Nursing and Healthcare-associated Infections

A recent evidence-based practice report sponsored by the Agency for Healthcare Quality and Research concluded that a relationship exists between lower levels of nurse staffing and higher incidence of adverse patient outcomes (*14*). Nurses' working conditions have been associated with medication errors and falls, increased deaths, and spread of infection (*15**–**30*) (Table). RN staffing levels have been associated with the spread of disease during outbreaks (*17**,**22**,**23**,**25**,**28*). However, increasing nurse-to-patient ratios alone is not adequate; more complex staffing issues appear to be at work. Many studies have found that the times of higher ratios of "pool staff" (i.e., nursing staff who were members of the hospital pool service or agency nurses) to "regular staff" (i.e., nurses permanently assigned to the unit) were independently associated with healthcare-associated infections (*16**,**17**,**21**,**27*). The skill mix of the staff, that is, the ratio of RNs to total nursing personnel (RNs plus nurses' aides), is also related to healthcare-associated infections; increased RN skill mix decreases the incidence of healthcare-associated infections (*20**,**29**,**30*). In a recent comprehensive review of the literature, the authors concluded that evidence of the relationship between nurses' working environment and patient safety outcomes, including healthcare-associated infections is growing. They also concluded that stability, skill mix, and experience of the nurse workforce in specific settings are emerging as important factors in that relationship (*31*).

**Table:** Summary of studies on nurse staffing and healthcare-associated infections^a^

Investigator	Sample	Findings
Outbreak investigations		
Anderson et al. (*17*)	36-bed neonatal ICU; 8 cases	During MRSA outbreak, 42% staff untrained, up to 62% from outside facility
Archibald et al. (*28*)	1 pediatric ICU; 43 patients	Decrease 2 infections/1,000 patient days for each unit increase in RN h: patient-day ratio^b^
Fridkin et al. (*25*)	230-bed VA center; 170 patients	Patient-nurse ratio increased during BSI outbreak^c^
Harbarth et al. (*22*)	15-bed neonatal ICU; 8 cases	*Enterobacter cloacae* outbreak terminated after decrease workload
Vicca (*23*)	1 adult unit; 50 cases	MRSA^b^ cases associated with increase workload, decrease RN-patient ratio
Prospective studies		
Alonso-Echanove et al. (*16*)	8 ICUs; 4,535 patients	Float RNs >60% central venous catheter days increased risk for BSI^d^
Haley et al. (*26*)	85-bed neonatal ICU; 76 infants	MRSA infections increased within 1 month of worsening workload^c^
Robert et al. (*21*)	20-bed surgical ICU; 28 cases	BSI associated with lower regular nurse-patient and higher pooled staff-patient ratios^b^
Retrospective studies		
Amaravadi et al. (*19*)	32 hospitals; 353 patients	Night nurse-patient ratio <1:2 associated with pneumonia^c^ and BSI^c^
Arnow et al. (*27*)	1 burn unit; 147 patients	New cases MRSA^b^ paralleled number of overtime h and number of shifts by outside staff
Knauf et al. (*30*)	502 hospitals	Pneumonia,^c^ postoperative infection,^c^ UTI^c^ associated with low RN h and skill mix
Kovner et al. (*15*)	530–570 hospitals; 10 states	Increase nurse h per adjusted patient day associated with decreased pneumonia^c^
Kovner & Gergen (*24*)	589 hospitals; 1,993 patients	Increase RN FTEs associated with decreased UTI^b^ and pneumonia^b^
Lichtig et al. (*20*)	1,575 hospitals	Pneumonia,^b^ postoperative infection,^b^ UTI^b^ associated with low RN skill mix
Needleman et al. (*29*)	799 hospitals; 6,180,628 patients	Higher proportion RN h, higher RN h per day resulted in decreased UTI^b^
Stegenga et al. (*18*)	44-bed pediatric unit; 2,929 admissions	<10.5 nurse h per patient day resulted in increased gastrointestinal infections^c^

^a^HAI, healthcare-associated infections; RN, registered nurse; MRSA, methicillin-resistant *Staphylococcus aureus*; BSI, bloodstream infection; UTI, urinary tract infection; VA, Veterans Administration; ICU, intensive care unit; FTE, full-time equivalent. ^b^Significant at <0.005. ^c^Significant at <0.05. ^d^Significant at 0.01.

## Nurses' Work and Occupational Exposure to Infectious Disease

All healthcare workers face a wide range of hazards on the job, including blood and body fluid exposure as well as musculoskeletal injuries related to ergonomic hazards from lifting and repetitive tasks; nursing personnel often experience these hazards most frequently (*32*). In 2001, U.S. hospitals reported 293,600 nonfatal occupational injuries and illnesses among their personnel. Among the eight private U.S. industries with >100,000 injuries and illnesses annually, the number of cases of nonfatal injury or illness in hospitals is the second highest; and the incidence rate of injuries and illnesses per 100 fulltime workers employed in nursing and personal care facilities is 13.5; by contrast, the national average is 1.8. In 2001, nursing aides and orderlies reported the highest number of occupational injuries that resulted in days away from work of any service industry (70,300); RNs had the second highest number (24,400) (*33*).

Work-acquired infectious diseases are among the risks all healthcare workers face; and bloodborne pathogens figure prominently among these. Occupational exposure to blood and body fluids is well documented among healthcare workers. Annual exposure prevalence rates range from <10% to 44%, depending on the occupational subgroup (*34*). Every year, approximately 600,000–800,000 occupational needlestick injuries occur in the United States (*34*). In a study of 60 U.S. hospitals in a 4-year period, nurses were the most likely to experience a blood or body fluid exposure (Figure) (*34*). Most exposures involve percutaneous injuries (e.g., needlesticks), although mucocutaneous (e.g., spray or splashes to the eyes or mouth) and direct contact of infected blood with nonintact skin are also routes of exposure. These potential infections, like healthcare-associated infections, also appear to be tied to nurses' working conditions. In a cross-sectional study of >1,500 nurses employed on 40 units in 20 hospitals, poor organizational climate and high workloads were associated with 50% to 200% increases in the likelihood of needlestick injuries and near-misses among hospital nurses (*3*).

**Figure:**
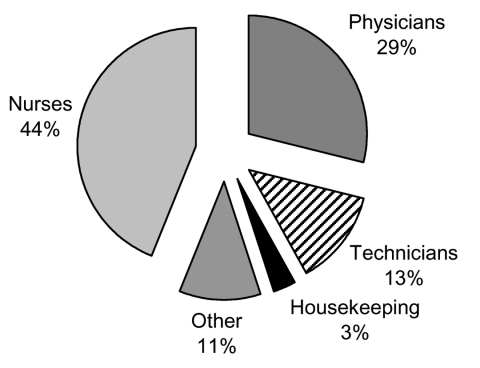
Blood and body fluids' exposure by personnel category. Source: National Institute for Occupational Safety and Health (*34*).

Emerging infectious diseases and outbreaks of recognized contagious illnesses have highlighted other concerns about the safety of healthcare workers. For example, much of the worldwide severe acute respiratory syndrome (SARS) outbreak was hospital-based, and healthcare workers made up a large proportion of cases, accounting for 37% to 63% of suspected SARS patients in highly affected countries (*35*). In many countries, nurses were the largest single group affected by SARS (*36*). During the Toronto outbreak, patient care activities commonly conducted by critical care nurses, such as manipulating oxygen masks and suctioning infected patients, were significantly associated with SARS infection (*37*). In the event of an influenza pandemic, healthcare workers would be susceptible. During an outbreak of parainfluenza in a intermediate care nursery, 16 (25%) of 65 staff members reported symptoms of respiratory illness (*38*). These threats to safety of the nurse and other essential healthcare workers are of concern for many reasons.

First, a trained, qualified healthcare workforce is necessary to respond and care for the public in the event of an outbreak. Staffing issues and hospital organization problems are believed to have complicated the containment of the SARS crisis in Toronto. Staffing shortages, especially of nurses, have been identified as one of the major factors expected to constrain hospitals' ability to deal with possible future threats (*4*). Without adequate numbers of trained hospital employees to implement effective infection control procedures, such as hand hygiene and proper isolation procedures, emergency departments and hospital wards can easily become the venues where the spread of epidemics occurs.

Second, the perception of unsafe working conditions both for the patient and the worker may actually hinder recruitment and retention of qualified staff. In a American Nurses Association survey of RNs (N = 7,353), 88% of respondents reported health and safety concerns related to work, 75% felt the quality of nursing care had declined in their work setting in the past 2 years, and 92% of those respondents related these concerns to inadequate staffing. Furthermore, >70% of respondents indicated concerns about the acute and chronic effects of work stress and overwork, concerns about a disabling back injury (60%), and fear of contracting HIV or hepatitis from a needlestick injury (45%). Nurses reported that these health and safety concerns influence their decision to continue working in the field of nursing and the kind of nursing work they choose to perform. Because of these concerns, nearly 55% of the nurses surveyed would not recommend the nursing profession as a career for their children or friends. Although the results of this survey may not be generalizable to all nursing personnel because of the nonprobability sampling method and inclusion of only RNs, the results suggest that concern over safety may be contributing to hospital personnel shortages and hindering recruitment efforts. Dissatisfaction, burnout, and concerns about quality of care are reportedly common among hospital nurses in five other industrialized countries (*39*).

## Gaps in Current Knowledge

Barring unprecedented growth in the nursing workforce or unforeseen new forces in health care that intervene to reduce burden of care in society, the numbers of nurses will not keep pace with the demand for services. In the coming decades, we face the prospect of fewer professionals and more unlicensed workers in the healthcare workforce. Decisions will have to be made about how hospitals will safely adapt to this situation. At this time, little evidence exists on what constitutes a safe and efficient labor force mix. Therefore, the general impact of nurse working conditions needs to be examined. First, longitudinal studies that track change in infection rates and other untoward incidents over time, under different working conditions, and with different staffing models are essential. Second, researchers need to study how the actual care received by patients varies under different staffing conditions at the bedside so that a better understanding of the impact of work environments at the point of care can be gained. Finally, since costs of care increase when patients have adverse outcomes (*40*) and nurses' working conditions affect outcomes, better working conditions could arguably save the healthcare system money. However, the cost-benefit ratio is not known and economic analyses, which include costs related to training, recruitment, and retention, need to be conducted.

## Implications for Policy

Policy solutions for nurse staffing fall into two general categories: 1) incentives and funding for various parties to increase the supply of nurses and 2) employer and hospital regulatory approaches. Although scholarships, loan forgiveness schemes, and funding of new nursing school student slots may be helpful, these policies are unlikely to overcome the long-standing, complex nature of the difficulties in recruiting sufficient newcomers to the nursing profession and then retaining a qualified workforce.

In the United States, regulatory approaches by the states have included prohibiting mandatory overtime for nurses (nine states with regulations), holding hospitals accountable for developing and implementing valid staffing plans (seven states), and setting minimum staffing ratios (one state). Regulating minimum nurse-patient ratios has received much attention, despite critiques from the hospital industry that insufficient data exist to credibly set minimum safe staffing levels. California was the first state to implement hospitalwide minimum nurse-patient ratios. The effects of this regulation need to be carefully examined. Although nursing services are positively correlated with patient outcomes, controversy exists over what constitutes an optimal staffing ratio, and little empirical evidence is available on which to base these decisions.

Staffing levels for bedside nurses are not the only critical resource involved in decreasing risks for healthcare-associated infections, occupational injuries, and infections. Also important is determining the critical mass of infection control and occupational health professionals needed for surveillance, identification of departures from sound practices, and ongoing education of healthcare workers. Policies aimed at ensuring the availability of training programs on all aspects of patient and worker safety are needed, as is the availability of appropriate supplies to prevent unnecessary infections among patients and nurses.

## Conclusions

Nursing is a predominately female occupation in which the working conditions are often poor. Such conditions contribute to recruitment and retention problems. Together with demographic changes, the result is a shortage of qualified nurses. Mounting evidence demonstrates that the lack of an adequate supply of qualified nurses is a global public safety issue that may require a multipronged policy approach. Monitoring and improving the working conditions of nurses are likely to improve the quality of health care by decreasing the incidence of many infectious diseases, assisting in retaining qualified nurses, and encouraging men and women to enter the profession. Further research is needed to understand how best to protect the patient as well as the healthcare worker. Changes in the workforce will have implications for infectious disease, infection control, and occupational health professionals with a need for much more thorough training of nonprofessionals in critical practices.
